# Hawkinsinuria With Direct Hyperbilirubinemia in Egyptian-Lebanese Boy

**DOI:** 10.3389/fped.2019.00069

**Published:** 2019-03-29

**Authors:** Hassan El Khatib, Bilal Asaad, Aisha Zaylaa, Farah Awad, Mariam Sbeity, Sirin Mneimneh, Georges Haber, Zeina Naja, Mariam Rajab

**Affiliations:** ^1^Department of Pediatric, Makassed General Hospital, Beirut, Lebanon; ^2^Orthopaedic Surgery, Mount Lebanon Hospital, Beirut, Lebanon

**Keywords:** hawkinsinuria, tyrosine, direct hyperbilirubinemia, HPD, heterozygous mutation

## Abstract

Tyrosinemia type III is the rarest type of tyrosinemia, because of a mutation in 4-OH-phenylpyruvate dioxygenase (HPD). This causes two different types of diseases with different modes of inheritance: tyrosinemia type III and hawkinsinuria. Hawkinsinuria is an autosomal dominant disease, which presents a failure to thrive and metabolic acidosis; however, the liver is not affected. P.A33T heterozygous mutation was reported by Tomoeda et al. to cause hawkinsinuria. This case report will present the first case of an Egyptian-Lebanese male who developed direct hyperbilirubinemia and was found to have tyrosinemia type III, due to elevated tyrosine levels in the blood and tyrosine derivatives in the urine, but genetic testing revealed a P.A33T heterozygous mutation, a cause of hawkinsinuria.

## Background

Tyrosine is produced internally as a metabolite of phenylalanine. Tyrosine aminotransferase (TAT) is an enzyme found in the liver that catalyzes tyrosine into 4-OH-phenylpyruvate, that is then broken down to homogentisate via 4-OH-phenylpyruvate dioxygenase (HPD). Homogentisate dioxygenase catalyzes the conversion of HPD to maleylacetoacetate and then to fumarylacetoacetate hydrolase (FAH) by maleylacetoacetate. Finally, the end product of fumarate and acetoacetate through FAH (1) is produced. Tyrosinemia is defined as the elevation of the tyrosine level in the blood, and there are three types of tyrosinemia (I-III). The rarest type is type III, which is the result of mutations in the HPD gene (12q14-qter) encoding 4-hydroxyphenylpyruvate dioxygenase (4-HPPD). These mutations in the HPD locus are related to two different diseases: tyrosinemia type III, which is inherited as an autosomal recessive trait, and hawkinsinuria, which is transmitted in an autosomal dominant form (2). Tyrosine accumulation in body fluids and the excessive excretion of its derivatives (4-hydroxyphenylpyruvic acid (4-HPP), 4-hydroxyphenyllactic acid (4-HPL), and hydrophenylacetic acid) into urine, characterize tyrosinemia type III (3). The enzyme is found in the liver and kidney, but it causes neurologic manifestations such as ataxia, intellectual disability and seizures, while hawkinsinuria causes metabolic acidosis and failure to thrive. This report will present a case of a 1-month-old Egyptian-Lebanese male who developed direct hyperbilirubinemia. Metabolic workup revealed tyrosinemia type III, and genetic testing confirmed the diagnosis of hawkinsinuria.

## Case Presentation

After 30 weeks and 3 days of gestation, male baby was born, to a G2P1A1 Lebanese mother and an Egyptian father. The course of pregnancy was complicated by gestational hypertension. The baby was born via an urgent C-section, due to preeclampsia with APGAR score 8, at the 1st minute and score 9 at the 5th minute of life. Birth weight = 1,390 g. The baby developed respiratory distress after birth and was admitted to the Neonatal intensive care unit (NICU). He was found to have hyaline membrane disease, after which two doses of surfactant were administered and he also required a mechanical ventilator. At day 23 of life, he developed enterococcus bacteremia, for which he received a 10 day course of Antibiotics (Vancomycin). At day 36 of life, he developed an enterobacter UTI as well as direct hyperbilirubinemia on the same day, with total bilirubin levels equaling 6.5 mg/dL (Normal: < 2 mg/dL) and direct bilirubin levels equaling 5.7 mg/dL (Normal: < 0.6 mg/dL), SGPT 111 U/L (Normal: 13–45 U/L), GGT 71 U/L (Normal: 13–147 U/L), alkaline phosphatase 738 U/L (Normal: 150–420 U/L), alpha-1 antitrypsin was normal 123 ( Normal: 90–200 mg/dL). He received a 7 day course of antibiotics (cefotaxime). After 1 week of treatment his bilirubin (T/D) increased to 6.5/5.7 mg/dL.

Direct hyperbilirubinemia persisted with bilirubin (T/D) 9.4/8.23 mg/dL, and high SGPT 149 U/L with borderline hypoglycemia, while the patient was on a Preemie formula (Pre Nursie).

After extensive revision of the differential diagnosis of direct hyperbilirubinemia, either a metabolic disease or cystic fibrosis remained as a possible diagnosis. At 50 days of life, DNA for cystic fibrosis tested negative, while plasma amino acids showed an elevated level of tyrosine at the same time: 263 μmol/L (Normal: 55–147 μmol/L). Urine organic acid showed elevated 4 hydroxyphenylacetic acid, 4 hydroxyphenylpyruvate acid, and 4 hydroxyphenylacetic acids. Alpha-fetoprotein was more than 121,000 ng/dL. HPD genetic studies showed the p.A33T (c.97G > A), which was detected in a heterozygous state. Genetic testing was not done for the parents. The patient was started on a Tyr-free formula (Tyr-Anamix-Infant SHS, UK), and frequent follow ups revealed a decrease in the Tyrosine level to 16 μmol/L (Normal: 55–147 μmol/L) after 30 days of initiating the special formula, and a subsequent decrease in bilirubin (T/D) to 5/2 mg/dL (Table 1) as well as some weight gain, reaching 2.8 Kg (Figure 2) after 45 days.

**Table 1:** Lab results.

	**Day of life 36**	**Day of life 49**	**Day of life 79**
SGPT (U/L)	111	149	
Bilirubin (T/D) (mg/dL)	6.5/5.7	9.4/8.23	5/2
Tyrosine (μmol/L)		263	16

## Discussion

Conjugated hyperbilirubinemia, generally known as neonatal cholestasis, that occurs in the newborn period or shortly thereafter, is defined as increased direct bilirubin more than 1.0 mg/dL (17.1 μmol/L) if the total serum bilirubin is < 5.0 mg/dL (85.5 μmol/L), or more than 20 percent of the total serum bilirubin, if the total serum bilirubin is >5.0 mg/dL (85.5 μmol/L) (4). Multiple etiologies cause conjugated hyperbilirubinemia in infancy, but mainly include extrahepatic biliary atresia (EHBA) (25.9%) and idiopathic neonatal hepatitis (INH) (26%) (5). In premature infants, cholestasis more frequently results from total parenteral nutrition (TPN) (6.4%) or sepsis (11.4%). Less common etiologies of infantile conjugated hyperbilirubinemia included alpha-1 antitrypsin deficiency (4.1%), perinatal hypoxia/ischemia (3.7%) (5). Metabolic diseases accounted for 4.4% (74 out of 1,692 subjects) as studied by Gottesman et al. (4, 5). Out of the 74 subjects found to have a metabolic disease by Gottesman et al. six subjects had tyrosinemia, accounting for 8.1% (5).

In 1975 Danks et al. reported a case of a baby girl who at the age of 20 weeks, presented metabolic acidosis and poor weight gain when feeding changed from breast milk to a higher protein formula (6). A low protein diet cleared the severe tyrosyluria and mild tyrosinemia, and also corrected the acidosis (6). However, restoration of growth required a normal protein intake with more reduced amounts of phenylalanine and tyrosine, to restore growth (6). There was no signs of liver disease or any problem in mental development (6). A new sulfur amino acid, called hawkinsin, which was identified as (2-L-cysteine-S-Cyl-1,4-dihydroxycyclohex-5-en-1-yr)-acetic acid, was detected in the urine of the same girl with prolonged tyrosinemia and in her mother, as stated by Niederwieser et al. (7). They claimed that an intermediate in the reaction of 4-hydroxyphenylpyruvate hydroxylase was the source of hawkinsin, and that the girl and her mother were heterozygous for a default in this hydroxylase system (7).

**Figure 1:**
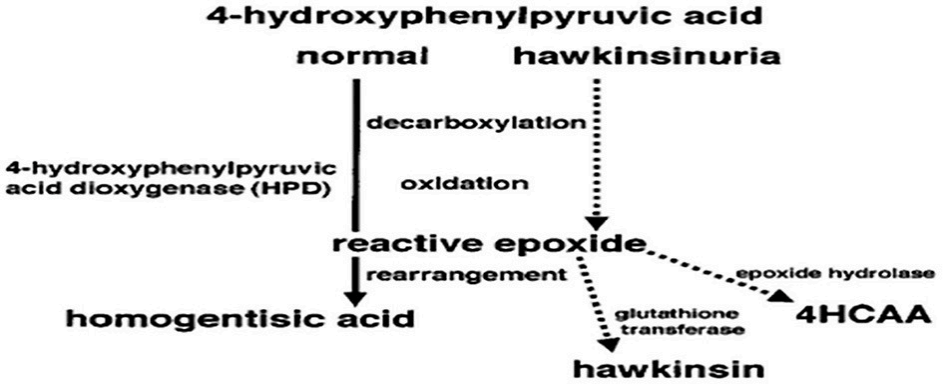
The reaction of 4-hydroxphenylpyruvic acid to homogentisic acid catalyzed by the enzyme 4-hydroxphenylpyruvic acid dioxygenase (HPD). Dotted lines show hypothesis of how, in hawkinsinuria, an enzyme capable of decarboxylation and oxidation will produce a reactive epoxide. If the enzyme cannot perform the final step of rearrangement to produce homogentisic acid, the reactive epoxide could then react with other cytoplasmic components of hawkinsin and hydroxycyclohexylacetic acid (4HCAA) (9).

The HPD gene is on 12q24 and has 14 exons and is activated in the cytoplasm where the enzyme is primarily expressed in the liver and kidney (8). Its mechanism of action is complicated and includes many steps, starting with a decarboxylation, then an oxidation, followed by rearrangement to form a homogentisic acid. However, the reactive epoxide would then dissociate from the enzyme and react with cytoplasmic components such as glutathione (to form hawkinsin) or water [to form Cis- and trans-hydroxycyclohexylacetic acids (4HCAA)] if the patient had an abnormal enzyme that can perform both decarboxylation and oxidation, but is not capable of catalyzing the final step of rearrangement (Figure 1) (9). Affected infants start to excrete 4HCAA isomers at an older age, however considerable amounts of 4HCAA isomers were detected in the urine of older affected persons (10). An epoxide hydrolase reaction of the intermediate epoxide, with later reduction and then detoxification of the reactive epoxide may be the source of these isomers (9). During infancy, this mechanism for detoxification may not be active. It will be activated when epoxide hydrolase amounts increase. Primarily, the diseased infants may be dependent on glutathione for detoxification, and symptoms may not appear until the protein load becomes high. Workup applied on family members of hawkinsinuria revealed that this disease is transmitted in a dominant way (7, 11). It has been suggested that breast milk may protect a patient from symptoms, as symptoms only started to appear when breast milk was changed to a formula. The reason for this change may be due to the lower level of protein found in breast milk, compared to a formula (9).

**Figure 2:**
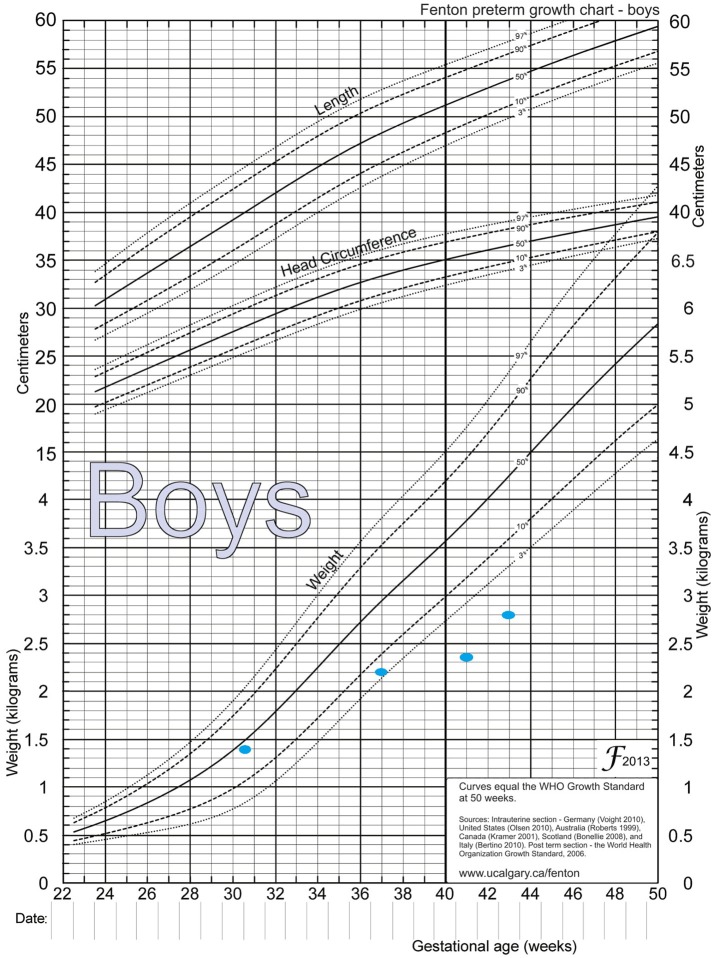
Fenton preterm growth chart, showing weight catchup. Birth weight = 1.39 Kg, between 36−37 weeks = 2.2 Kg, between 41−42 weeks = 2.35 Kg, and at 44 weeks = 2.8 Kg.

The patient studied in this case report was found to have hawkinsinuria as confirmed by genetic testing where a p.A33T (c.97G > A) heterozygous mutation was detected, same as that reported by Tomoeda et al. An incompletely ineffective enzyme that is able to perform decarboxylation and oxidation, but unable to perform rearrangement may be the result of this mutation (9). In heterozygotes, symptoms are caused due to the formation of reactive epoxide resulting from the translation of an abnormal enzyme due to this mutation, even if present in one copy (9).

## Conclusion

To our knowledge this is the first case of direct hyperbilirubinemia that after extensive workup and by ruling out common causes, ended up revealing the case of hawkinsinuria, in an Egyptian-Lebanese baby. This is the first case of hawkinsinuria diagnosed in the Middle East.

## Consent

Written informed consent was obtained from the patient's family for publication of this case report.

## Author Contributions

All authors listed have made a substantial, direct and intellectual contribution to the work, and approved it for publication.

### Conflict of Interest Statement

The authors declare that the research was conducted in the absence of any commercial or financial relationships that could be construed as a potential conflict of interest.
